# LINC00957 Acted as Prognostic Marker Was Associated With Fluorouracil Resistance in Human Colorectal Cancer

**DOI:** 10.3389/fonc.2019.00776

**Published:** 2019-08-21

**Authors:** Li Hua Zhang, Long Hai Li, Peng Fei Zhang, Yan Fei Cai, Dong Hua

**Affiliations:** ^1^Affiliated Hospital of Jiangnan University, Jiangnan University, Wuxi, China; ^2^School of Pharmaceutical Science, Jiangnan University, Wuxi, China; ^3^Wuxi Medical College, Jiangnan University, Wuxi, China

**Keywords:** LINC00957, colorectal cancer, drug-resistance, survival, lincRNA, TCGA, GSEA

## Abstract

Colorectal cancer (CRC) is one of the most prevalent digestive tumors in China. Recent studies indicate that long intergenic non-coding RNAs (lincRNAs) play a crucial role in predicting survival for CRC patients. However, the novel lincRNA, LINC00957, is largely unclear in CRC. The purpose of the current study was to determine LINC00957 expression, assess its the clinical significance and explore the potential mechanism in CRC. The qRT-PCR was used to quantify the expression levels of LINC00957 in tissues and cell lines. Our research revealed that LINC00957 was significantly higher expression in CRC. In addition, the LINC00957 expression was associated with TNM stage and chemotherapy outcome, but age, gender, tumor size, histological grade, primary tumor location. CRC patients with high LINC00957 expression level showed poor overall survival (*P* = 0.002). Multivariate survival analysis indicated that LINC00957 was a prognostic factor for CRC patients (*P* = 0.010). Mechanically, inhibition of LINC00957 expression reversed 5-FU resistance by down-regulating P-gP. In summary, our study indicated that this novel lncRNA expression signature might be a useful biomarker of the prognosis and therapeutic target for CRC patients.

## Introduction

Colorectal cancer (CRC) has been reported to be one of the most common malignant tumors in the digestive system, with a high recurrence and cancer-related mortality rate worldwide (1). In China there were about 376,000 newly diagnosed colorectal cancer patients in 2015, leading to about 191,000 deaths (2). Despite wide application of standardized surgical treatment and better perioperative care, the treatment of CRC at early stages remains a challenge (3, 4). Therefore, there is an urgent need to seek for new biomarkers for early diagnosis and prognoses in patients with CRC and to develop potential targets for the therapeutic treatment. To better understand the mechanism of CRC, non-coding RNAs have been recently studied in CRC such as lncRNAs and lincRNAs. Long non-coding RNAs (lncRNAs) are aberrantly expressed in a broad spectrum of cancers, and they play key roles in promoting and maintaining tumor initiation and progression, demonstrating their clinical potential as biomarkers and therapeutic targets (5–8). Long intergenic non-coding RNAs (lincRNAs) are defined as autonomously transcribed non-coding RNAs longer than 200 nucleotides that do not overlap annotated coding genes, as future cancer biomarkers (9, 10). More and more lincRNAs have been found that they played important roles in CRC such as LINC00261, LINC01420 (11, 12). Recent studies indicate that long intergenic non-coding RNAs (lincRNAs) play a crucial role in predicting survival for CRC patients such as linc-UBC1 (13) and linc-UFC1 (14). However, the novel molecule, LINC00957, in CRC is still unclear.

In this study, we explore its function in CRC. Firstly, we analyzed lincRNA expression profiles in 440 CRC patients from The Cancer Genome Atlas (TCGA) and identified it was significantly associated with survival. Then we further examined the expression of LINC00957 in CRC tissues and cell lines compared to their matched adjacent non-tumor by qRT-PCR. Besides, we also analyzed the association between the LINC00957 expression and the clinic pathological characteristics of CRC patients. Mechanically, we explored that its function in 5-FU resistance was associated with multi-drug resistance relative genes. All data demonstrated that it was up-regulated in CRC, and was associated with TNM stage, chemotherapy outcome, but not with the age, gender, tumor size, histological grade, and primary tumor location. What's more, the high expression of LINC00957 suggested poor survival in CRC and involved in drug-resistance by regulating P-gp expression. Taken together, our study suggested that the upregulation of LINC00957 played a promoting role in CRC and it would be a potential biomarker for CRC progress and therapeutic target of CRC.

## Materials and Methods

### Patient Samples

one hundred and sixty pairs of CRC tissues and adjacent normal tissues involved in present study were collected from Affiliated Hospital of Jiangnan University. Informed consents to approve our use of these tissues in the research were obtained from all patients. Patients who postoperatively received 5-Fu-based first-line systematic chemotherapy were enrolled in the present study. The protocol was approved by Affiliated Hospital of Jiangnan University. All methods involving human patients were performed in accordance with the relevant guidelines and regulations of Affiliated Hospital of Jiangnan University. These specimens were confirmed by histopathological examination and immediately stored at −80°C until use.

Tumor assessment was performed after every 2 cycles of chemotherapy according to the Response Evaluation Criteria in Solid Tumors 1.1 (RECIST 1.1) criteria (22), and the assessment was classified as a complete response (CR), a partial response (PR), stable disease (SD) and progressive disease (PD).

### Cell Lines and Cell Culture

Human CRC cell lines (HCT8, SW480, SW620, LOVO, and HCT116) and normal human colon epithelial cells NCM460 were purchased from Nanjing Musai Bio-Tech Co, Ltd (Nanjing, China). Fluorouracil (5-Fu)-resistant HCT-8 cells (HCT-8/5-Fu) (KG333) was purchased from Keygen Biotech Co. Ltd. 5-Fu-resistant LoVo cells (LoVo/5-Fu) were derived by treating LoVo cells with stepwise increasing concentrations of 5-Fu (Jinyao Amino Acid Co. Ltd, Tianjin, China) over 6 months. 5-Fu-resistant CRC cells were cultured as we reported previously (15). All cells were cultured in DMEM (Life Technologies) supplemented with 10% fetal bovine serum (FBS; Invitrogen), 10% (100 μg/mL) penicillin and 100 U/mL streptomycin at 37°C with 5% CO_2_ in a humidified atmosphere.

### RNA Extraction and qRT-PCR Assay

Total RNA was isolated from CRC and matched adjacent tissues by using Trizol Reagent (Life Technologies), according to the manufacture's protocol. The concentration and purity of total RNA were measured on a nanodrop spectrophotometer (Thermo Scientific, USA). For qRT-PCR, RNA was reverse transcribed to cDNA by using a PrimeScript RT reagent Kit (Takara, Dalian, China). qRT-PCR was performed using a SYBR_Premix ExTaqII kit (Takara, Dalian, China) in the CFX96 Real-Time PCR Detection System (Bio-Rad, Hercules, CA, USA) to determine the relative expression levels of target genes. The reaction conditions was 95°C for 5 min, followed by 40 cycles of 95°C for 15 s and 60°C for 30 s. The 2^−Δ*Ct*^ method was used to calculate expression levels and 2^−ΔΔ*Ct*^ method was used to calculate relative expression level. GAPDH was used as an internal control (16). The sequences of qRT-PCR primers as follow Table 1.

**Table 1:** Sequences of gene-specific primers used for RT-PCR.

**Gene**	**Forward Primer (5′-3′)**	**Reverse Primer (5′-3′)**
LINC00957	TCAAGGGCGGAGCAAACATC	AGTTTGCAAAGCCTTCCTGTG
ABCA1	CAGGCTACTACCTGACCTTGGT	CTGCTCTGAGAAACACTGTCCTC
ABCG2	GTTCTCAGCAGCTCTTCGGCTT	TCCTCCAGACACACCACGGATA
ABCC1	CCGTGTACTCCAACGCTGACAT	ATGCTGTGCGTGACCAAGATCC
ABCC2	GCCAACTTGTGGCTGTGATAGG	ATCCAGGACTGCTGTGGGACAT
GAPDH	GCACCCTGGTCTGAGGTTAAAT	AGGAGTGGGAGCACAGGTAAG

### Cell Transfection

LOVO/5-FU and HCT8/5-Fu cells on 50–70% confluence were treated with LINC00957-siRNA1 (GGUGGAAGAGCUUGGGCGACA, UCGCCCAAGCUCUUCCACCUG), LINC00957-siRNA2 (UUAAACCUCCCAUCAUCUGUG, CAGAUGAUGGGAGGUUUAACU), LINC00957-siRNA3 (AUCAUAACAUGGUGAAAGGCA, CCUUUCACCAUGUUAUGAUGC) and nontargeting siRNA (si-NC), respectively. Cells were transfected with siRNA by using Lipofectamine 4000 (Invitrogen, Carlsbad, CA, USA) according to the manufacturer's instructions. The cells were harvested for further assays 48 h after transfection. Expression of LINC00957 were determined by Real-time PCR. Three independent experiments were performed, and the data are expressed as the mean ± standard error of the mean (SEM).

### CTB Assay

These cytotoxic effects of 5-FU were evaluated at 48 h with the CTB (celltiter-blue, cell viability assay) assay. Cells (1 × 10^5^cells/200 ul) were seeded in 96-well plates and placed in an incubator at 37°C for 12 h. The medium was then replaced with medium containing different concentrations of 5-FU. After 48 h, the medium of each well was added with 10 uL CTB. After incubation for 4 h, the plates were read on a microplate reader. Fluorescence density at 560 nm excitation and 590 nm emission was determined for statistical analysis. Three independent experiments were performed, and the data are expressed as the mean ± standard error of the mean (SEM).

### Western Blot Assay

Cells grown in 6-well plates were washed with cold PBS for three times and scraped off the culture dishes and treated with 200 ul RIPA buffer containing protease inhibitors and were then transferred to 5 ml tubes. Those tubes were remained in ice for 30 min to cells to be completely lysed. After a centrifugation at 13,500 rpm for 15 min under 4°C, the supernatants were collected and were subsequently denatured with 1X SDS protein loading buffer at 100°C in 10 min. Proteins were loaded on 12% SDS-PAGE gels and transferred onto PVDF membranes. The membranes blocked with 5% BSA in TBST at room temperature for 2 h and incubated with primary antibodies under 4°C overnight. The primary antibodies anti- ABCA1 antibody (1:1000, ab18180, abcam) and anti-GAPDH (1:5000, ab8245, abcam)were used to detect the expression of the proteins. The antigen-antibody complexes were visualized by an enhanced chemiluminescent reaction. Protein bands were analyzed using Image J software (National Institutes of Health, Bethesda, MD). Three independent experiments were performed, and the data are expressed as the mean ± standard error of the mean (SEM).

### Data Mining and Analysis

The LINC00957 gene expression in different tissues was from The Genotype-Tissue Expression (GTEx) project (https://gtexportal.org/).

Data in OncoLnc from the website (www.oncolnc.org, date to September, 2018) was been used. Then all data for individual cancers from TCGA were downloaded.

### Gene Set Enrichment Analysis (GSEA)

The RNA-sequencing V2 datasets and clinical data of STAD samples were downloaded using the Bioconductor/TCGA Biolinks function package from TCGA database (http://tcga-data.nci.nih.gov./tcga/). The STAD-associated gene clusters and pathways were identified in the c2.cp.kegg.v5.1.symbols.gmt data set from the Msig database using GSEA version 3.0. Enrichment analysis was performed with random combination number of 1,000 and false discovery rate (FDR) <0.05 as the criteria for significantly enriched genes. The gene sets were classified into the LINC00957 high- and low-expression groups based on its median expression level, and the effect of LINC00957 expression was analyzed.

### Statistical Analysis

The expression levels of LINC00957 in CRC tissues were analyzed by unpaired *t* test. The chi-square and *t* tests were performed to assess the relationship between LINC00957 expression and clinicopathological features. The Kaplan-Meier method and log-rank test were used to evaluate and compare the prognosis of CRC patient. Univariate and multivariate cox proportional hazards analyses were used to analyze the independent prognostic factors for survival in CRC patients. RStudio and GraphPad Prism software package were used to perform statistical analysis. Overall survival (OS) was calculated using the Kaplan-Meier method, and the results of the analysis were considered significant in a log-rank test if *P* < 0.05.

## Results

### LINC00957 Was Abnormal Expression in Different Tissues and Only Associated With Colon Cancer in Gastrointestinal Cancer

**Figure 1:**
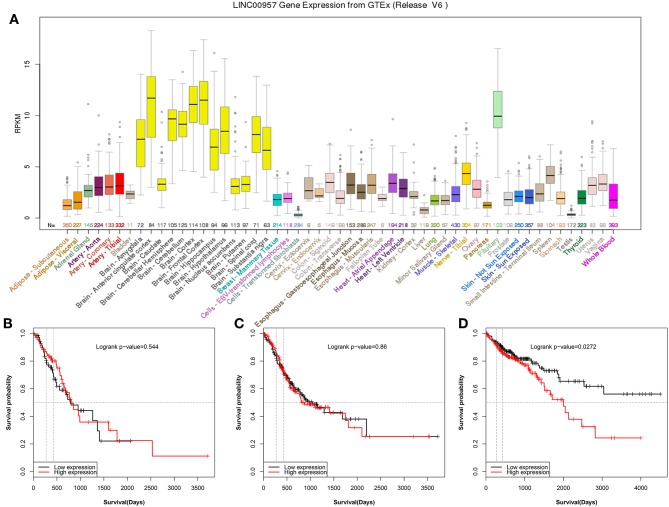
The gene expression of LINC00957 in different tissues and its relationship of Gastrointestinal cancer. **(A)** The gene expression of LINC00957 in different human tissues from GTEx. From the TCGA database, colon cancer patients with High LINC00957 expression (*n* = 220) also showed significantly shorter survival time than those with low LINC00957 expression (*n* = 220). The red curve represented high expression. **(B)** From the TCGA database, the expression of esophageal carcinoma was associated with overall survival (Logrank *P*-value = 0.544, *N* = 144). **(C)** From the TCGA database, the expression of stomach adenocarcinoma was associated with overall survival (Logrank *P*-value = 0.86, *N* = 378). **(D)** From the TCGA database, the expression of colon adenocarcinoma was associated with overall survival (Logrank *P*-value = 0.0272, *N* = 440).

In order to explore LINC00957 expression, GTEx database was used to present the gene expression in different human tissues. It was showed that LINC00957 was abnormal expression in different tissues as Figure 1A. In Gastrointestinal cancer, esophageal carcinoma and stomach adenocarcinoma was not associated with overall survival (*P* > 0.05, Figures 1B,C). However, colon adenocarcinoma was associated with overall survival (*P* < 0.05, Figure 1D).

### LINC00957 Was Upregulated in CRC Tissues and Cell Lines

**Figure 2:**
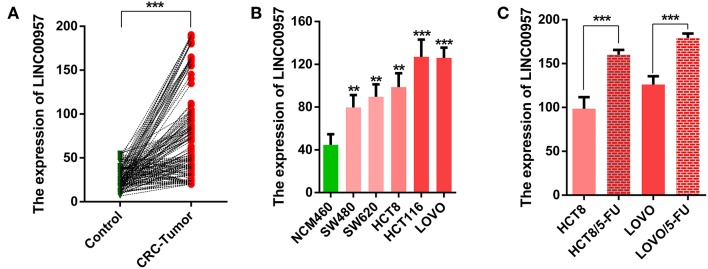
The relative expression level of LINC00957 in tissue samples and cell lines. **(A)** The green points represented normal tissues as control, while the red stood for CRC. A line connects two pionts (the expression levels of LINC00957 shown in **(A)** from non-tumor to CRC with an increased trend, suggesting that the expression level of LINC00957 was higher in CRC tissues than in the matched non-tumor tissues. **(B)** Expression levels of LINC00957 in CRC cell lines including (SW480,SW620,HCT8,LOVO, and HCT116) and the normal human colon epithelial cell line NCM460 was measured by qRT-PCR. **(C)** The relative expression levels of LINC00957 in CRC cell lines and their matched 5-FU resistant cell lines were detected by qRT-PCR. **P < 0.01, ***P < 0.001.

In this study, we firstly compared the expression of LINC00957 by qRT-PCR in 160 cases of CRC tissues and their matched adjacent non-tumor tissues. As shown in Figure 2A, we found that the expression of LINC00957 in CRC tissues were significantly higher than that in normal tissues (*P* < 0.001). We further examined its expression in the normal human colon epithelial cell line NCM460 and CRC cell lines (HCT8,SW480,SW620,LOVO and HCT116). As shown in Figure 2B, those data indicated that the expression of LINC00957 were also increased in CRC cell lines when compared with that in NCM460 cells. What's more, the expression of LINC00957 in 5-FU resistant cell lines were significantly upregulated as showed in Figure 2C. Those results showed that the expression of LINC00957 was aberrant in tissues and cell lines. It indicated that the expression was associated with CRC and drug resistance.

### LINC00957 Expression Was Associated With TNM Stage and Chemotherapy Outcome of CRC

To further reveal the role of LINC00957 in CRC, we analyzed the correlation between LINC00957 expression level and clinicopathological features. Patients with CRC were divided into high and low expression group by the mean expression level of LINC00957. Those data showed that the high expression group was significantly correlated with TNM stage (*P* = 0.040) and chemotherapy outcome (*P* = 0.026). However, the level of LINC00957 expression in CRC tissues was not associated with the age, gender, tumor size, histological grade, primary tumor location (all *P* > 0.05, Table 2). These findings suggested that the increased expression of LINC00957 was involved in the malignant progression and chemotherapy resistance of CRC patients.

**Table 2:** The correlation between LINC00957 expression and clinicopathological variables in CRC patients.

**Variable**	**Number**	**Low expression**	**High expression**	**x^**2**^ test**	***P*-value**
**AGE (YEARS)**					
<60	82	47	35	0.078	0.874
≥60	78	43	35		
**GENDER**					
Male	73	42	31	0.09	0.873
Female	87	48	39		
**TUMOR SIZE**					
<4cm	71	37	34	0.888	0.423
≥ 4cm	89	53	36		
**DIFFERENTIATION**					
Well/moderately	84	46	38	0.159	0.751
Poorly	76	44	32		
**PRIMARY TUMOR LOCATION**					
Colon cancer	78	41	37	0.84	0.426
Rectal cancer	82	49	33		
**TNM STAGE**					
I-III stage	116	71	45	4.212	**0.040**
IV stage	44	19	25		
**CHEMOTHERAPY OUTCOME**					
CR/PR	117	72	45	4.948	**0.026**
SD/PD	43	18	25		

Bold values indicate P < 0.05.

**Figure 3:**
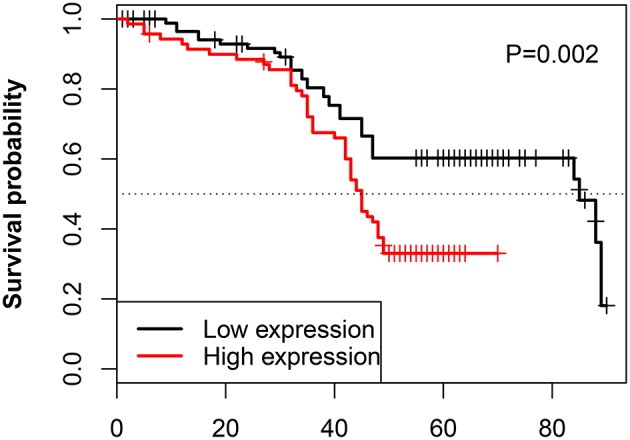
Correlation between LINC00957 expression and OS time in CRC patients. Kaplan-Meier postoperative survival curve for patterns of patients with CRC and LINC00957 expression. In our data, CRC patients with High LINC00957 expression (*n* = 80) showed shorter survival time than those with low LINC00957 expression (*n* = 80).

The relationship between the LINC00957 expression and the survival time of CRC patients was further analyzed. Our data showed that CRC patients with high LINC00957 expression appeared a worse prognosis when compared with those with low level in Figure 3 (*P* = 0.002). At the same time, we analyzed the relationship of LINC00957 expression levels and prognosis in CRC patients from TCGA database. LINC00957 expression showed shorter survival time than those with low LINC00957 expression in Figure 1D (*P* = 0.03). We then analyzed the factors of poor prognosis by univariate and multivariate cox proportional hazards analyses. In univariate overall survival (OS) analysis, we observed that LINC00957 expression (*P* < 0.001), TNM stage (*P* < 0.001), chemotherapy outcome (*P* = 0.014) and gender (*P* = 0.048) retained significance as prognostic factors (Table 3). In multivariate OS analysis, we showed that LINC00957 expression (*P* = 0.010) and chemotherapy outcome (*P* = 0.018) were an independent predictor of poor prognosis. Accordingly, our data demonstrated that high expression of LINC00957 could predicate a poor prognosis of patients with CRC.

**Table 3:** Univariate and multivariate analysis of prognostic factors of CRC.

**Variable**	**Univariate analysis**	**Multivariate analysis**		
	**HR (95%CI)**	***P*-value**	**HR (95%CI)**	***P-*value**
**Age** (<60 vs. 60>)	0.997 (0.981–1.012)	0.674		
**Gender** (Male vs. Female)	2.002 (1.009–3.989)	0.048		
**Tumer size** (<4cm vs. >4cm)	0.965 (0.909–1.026)	0.256		
**Histological grade** (Well/moderately vs. Poorly)	0.874 (0.639–1.195)	0.400		
**Primary tumor location** (colon vs. rectal)	0.846 (0.617–1.159)	0.297		
**TNM stage** (IV stage vs. I-III stage)	1.913 (1.339–2.734)	<0.001		
**Chemotherapy outcome** (SD/PD vs. CR/PR)	1.844 (1.283–2.650)	0.014	1.541 (1.066-2.228)	0.018
**LINC00957** (High vs. Low expression)	3.008 (2.051–4.412)	<0.002	2.718 (1.839-4.017)	0.010

### LINC00957 Expression Was Associated With Drug Resistance by Regulating P-gP

**Figure 4:**
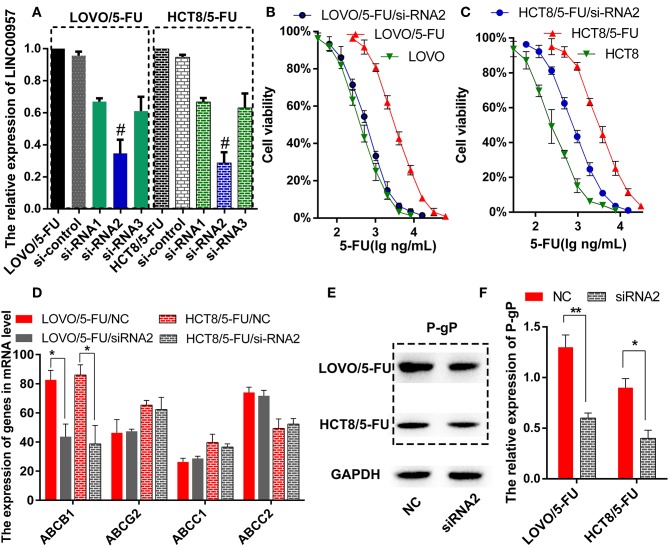
Inhibition of LINC00957 reversed 5-FU resistance by regulating P-gp expression. **(A)** In LOVO/5-FU and HCT8/5-FU cell lines, LINC00957 expression was examined by qRT-PCR in the situations of non-transfected, si-control, siRNA1, siRNA2 and siRNA3. ^#^stood for the best silent effect, **stood for *P* < 0.001. **(B)** Inhibition of LINC00957 by siRNA2 reversed 5-FU drug resistance in LOVO/5-FU cell lines. **(C)** Inhibition of LINC00957 by siRNA2 reversed 5-FU drug resistance in HCT/5-FU cell lines. **(D)** The relative multi-drug resistance genes(ABCB1, ABCG2, ABCC1, ABCC2) were examined in LOVO/5-FU and LOVO/5-FU/siRNA2,also in HCT8/5-FU and HCT8/5-FU/si-RNA2. The ABCB1 was significantly down-regulated by inhibition of LINC00957 in LOVO/5-FU and HCT8/5-FU. *stood for *P* < 0.05. **(E)** The expression of P-gp, which is coded by ABCB1,was detected by western blot. **(F)** The expression of P-gp was significantly down-regulated by inhibiting LINC00957 (siRNA2) than normal as control (NC) in LOVO/5-FU and HCT8/5-FU cell lines, respectively.

The two 5-Fluorouracil-resistant model cell lines (LOVO/5-FU and HCT8/5-FU) were used to study the resistance mechanism of LINC00957. Firstly, three pairs of silence primers of LINC00957 were designed to reduce the expression of LINC00957 in both resistant cells. As showed in Figure 4A, the silence effect of siRNA2 in these two cells was the best than siRNA1 and siRNA3, and the result of siRNA2 was remarkably lower expression of LINC00957 comparted with non-silence (*P* < 0.001). According to the CTB experiment, the IC_50_ of LOVO was 428.5 ng/mL (LogIC50 = 2.632 ± 0.051); the IC_50_ of LOVO/5-FU was 1621 ng/mL (LogIC50 = 3.210 ± 0.048); the IC_50_ of LOVO/5-FU/si-RNA2 was 590.1 ng/mL (LogIC50 = 2.771 ± 0.061). The IC_50_ of HCT was 409.8 ng/mL (LogIC50 = 2.613 ± 0.052); the IC_50_ of HCT/5-FU was 3064 ng/mL (LogIC50 = 3.486 ± 0.045); the IC_50_ of HCT/5-FU/si-RNA2 was 941.3 ng/mL (LogIC50 = 2.974 ± 0.096). As shown in Figures 4B,C, inhibition of LINC00957 significantly reversed the 5-FU resistance in both model cell lines. By low-throughput screening of the drug-resistant relative genes, the transcription level of ABCB1 gene was significantly down-regulated after interfering with RNA (Figure 4D). At the same time, the expression of P-gP in both cell lines were also significantly down-regulated at the protein level ABCB1 (Figures 4E,F). In clinical samples, LINC00957 was positively associated with P-gP both in in transcription and protein levels (all *P* < 0.05, Table 4). These results indicated that LINC00957 was involved in P-gP mediated chemo-resistance.

**Table 4:** The correlation of LINC00957 with P-gp both in transcription and protein levels in clinical samples.

**Variable**	**Number**	**LINC00957 ^**high**^**	**LINC00957 ^**low**^**	**x^**2**^ test**	***P*-value**
ABCB1 ^high^	62	34	28	4.349	0.037
ABCB1 ^low^	98	36	62		
P-gP ^high^	69	38	31	5.536	0.019
P-gP ^low^	91	32	59		

## Discussion

Colorectal cancer ranks second in terms of mortality and third in terms of incidence, accounting for about 10% new colorectal cancer cases and deaths in 2018 (5, 17). Therefore, it is necessary to identify new molecular targets for the diagnosis, prognosis, and treatment of CRC (18). LincRNAs play vital roles in many biological processes and numerous studies have figured out that aberrant expression of lincRNAs is closely associated with the occurrence and development of malignant tumors (19–21). For instance, lincRNA DQ786243 contributed to proliferation and metastasis of colorectal cancer both *in vitro* and *in vivo* (22); GHET1 and TUG1 promoted colon cancer progression (23, 24); GAS5 contributed to lymphatic metastasis in colorectal cancer (22); Linc00152 and LincRNA-ROR hold prognostic value (15, 25). LncRNAs were reported to be involved in CRC stem cells, and several individual lncRNAs were connected to Wnt signaling through other mechanisms (26). All those reports showed the missing non-coding lincRNAs played vital roles in CRC. However, the role of linc00957 in CRC is still unknown up to now. In order to find interested lincRNA, we analyzed some lincRNAs expression levels using OncoLnc (25), linking TCGA survival data to mRNAs, miRNAs, and lncRNAs (27). We found that patients with higher LINC00957 expression level had dramatically shorter OS in CRC, while in ESAD and STAD are not. it might be that LINC00957 was prognostic marker special for CRC. Therefore, it was necessary to explore its function in CRC.

Firstly, we explored whether LINC00957 was abnormally expressed in CRC. RT-PCR analysis was used to detect the expression of LINC00957 in 160 cases of the cancer and adjacent tissues. The results showed that LINC00957 was highly expressed in the cancer, and the same result was also found in cell lines. These data indicated that LINC00957 was associated with CRC. Secondly, we explored the correlation between the expression of LINC00957 and its clinicopathological variables. These results revealed that its high expression was significantly associated with TNM advanced stage, poor chemotherapy outcomes and worse prognosis. It revealed that the expression of LINC00957 was involved in tumor progression and multidrug resistance, and could be an independent prognostic indicator. Some references had reported that LINC-VLDLR and LINC-ROR were associated drug resistance (28, 29). Therefore, we speculated that LINC00957 might affect poor prognosis through inducing chemo-resistance. To test the hypothesis, we performed two 5-FU resistant cell lines (LOVO/5-FU and HCT8/5-FU) as chemo-resistance model cells. It was found that LINC00957 was remarkably higher expression in these chemo-resistance model cells than relative sensitive cell lines (LOVO and HCT8).Inhibition of its expression enhanced sensitivity and reversed 5-FU resistance. In the mechanism, we speculated that the relative multi-drug genes were associated with LINC00957 expression. P-gp(known as multidrug resistance protein 1 and encoded by the ABCB1 gene) efflux activity is capable of lowering intracellular concentrations of otherwise beneficial compounds, such as chemotherapeutics and other medications, to sub-therapeutic levels (30). Consequently, P-gp overexpression is one of the main mechanisms behind decreased intracellular drug accumulation and development of multidrug resistance in human multidrug-resistant (MDR) cancers (31). Through low throughput screening, only P-gP expression was down-regulated after inhibiting the expression of LINC00957.It indicated that LINC00957 might reverse 5-FU resistance by regulating P-gP expression. To further explore this mechanism, we enriched KEGG pathway by GSEA from TCGA database. The Notch signaling pathway was significantly enriched in LINC00957^hi^ group. It suggested that LINC00957 might induce multidrug resistance by activate Notch signaling pathway. Recently, emerging evidences suggest that Notch signaling pathway is one of the most important signaling pathways in drug-resistant tumor cells (32). The activated Notch signaling contributes to resistance in enzalutamide-resistant prostate cancer cells (33). Inhibition of Notch signaling reverses docetaxel resistance in prostate cancer (34).

These findings demonstrated that the long non-coding RNA 00957 (LINC00957) expression as a novel prognostic biomarker in colorectal cancer (CRC). Aberrant expression of LINC00957 was associated with CRC in GTEx dataset, and his upregulation was confirmed by qRT-PCR in patients' tumor and paired normal tissues. Expression of LINC00957 was significantly associated with advanced TNM stage, poor chemotherapy outcomes, and worse prognosis. Thus, we speculated that LINC00957 was involved in tumor progression and development of multidrug resistance. The latter hypothesis was partially demonstrated using two 5-Fluorouracil-resistant model cell lines (LOVO/5-FU and HCT8/5-FU), in which the silencing of LINC00957 was able to reverse 5-FU resistance, potentially through P-gP modulation. Beyond the functional study using knockdown experiments, this study also shows the potential role of the linc00975 in drug resistance. All these data together suggest a role of LINC00957 as oncogenic gene in CRC. Overall, this study is novel and well-performed, representing a good starting point for the exploration of the role of LINC00975 in CRC. The results obtained from CRC patients' analysis are a reasonable base for further experiments.

This research had some limitations. Firstly, the study is very linear, clear and simple. However, the evidences supporting the involvement of LINC00975 overexpression in the development of a drug-resistance phenotype are poor. Additional and more pround experiments are needed to sustain with mechanistic evidences, the designation of LINC00975 expression as a potential therapeutic target for CRC. Secondly, all experiments have been performed well and reasonably. Although the functional mechanism of linc00975 in CRC development and drug-resistance have not been investigated, it could be continued in the further studies.

## Data Availability

The raw data supporting the conclusions of this manuscript will be made available by the authors, without undue reservation, to any qualified researcher.

## Author Contributions

DH and LZ designed the study. LZ and LL developed the methodology, performed the analysis, and wrote the manuscript. PZ, YC, and LL collected the data, cultured cell lines and did PCR, CTB, Western blot assays.

### Conflict of Interest Statement

The authors declare that the research was conducted in the absence of any commercial or financial relationships that could be construed as a potential conflict of interest.

## References

[1] Sinicrope FA. Lynch syndrome-associated colorectal cancer. N Engl J Med. (2018) 379:764–73. 10.1056/NEJMcp1714533 30134129

[2] Chen W Zheng R Baade PD Zhang S Zeng H Bray F. Cancer statistics in China, 2015. CA Cancer J Clin. (2016) 66:115–32. 10.3322/caac.21338 26808342

[3] Dienstmann R Mason MJ Sinicrope FA Phipps AI Tejpar S Nesbakken A. Prediction of overall survival in stage II and III colon cancer beyond TNM system: a retrospective, pooled biomarker study. Ann Oncol. (2017) 28:1023–31. 10.1093/annonc/mdx052 28453697 PMC5406760

[4] Suenaga M Akiyoshi T Shinozaki E Fujimoto Y Matsusaka S Konishi T. A feasibility study of capecitabine and oxaliplatin for patients with stage II/III colon cancer -ACTOR Study. Anticancer Res. (2018) 38:1741–7. 10.21873/anticanres.12410 29491111

[5] Tutino V Defrancesco ML Tolomeo M DE Nunzio V Lorusso D Paleni D. The expression of riboflavin transporters in human colorectal cancer. Anticancer Res. (2018) 38:2659–67. 10.21873/anticanres.12508 29715086

[6] Lea MA Kim H Des BC. Effects of biguanides on growth and glycolysis of bladder and colon cancer cells. Anticancer Res. (2018) 38:5003–11. 10.21873/anticanres.12819 30194144

[7] Tei M Otsuka M Suzuki Y Kishi K Tanemura M Akamatsu H. Safety and feasibility of single-port surgery for colon cancer in octogenarians. Anticancer Res. (2018) 38:2967–2972. 10.21873/anticanres.12547 29715125

[8] Dai W Feng Y Mo S Xiang W Li Q Wang R. Transcriptome profiling reveals an integrated mRNA-lncRNA signature with predictive value of early relapse in colon cancer. Carcinogenesis. (2018) 39:1235–44. 10.1093/carcin/bgy087 29982331

[9] Wang Y Ding X Hu H He Y Lu Z Wu P. Long non-coding RNA lnc-PCTST predicts prognosis through inhibiting progression of pancreatic cancer by downregulation of TACC-3. Int J Cancer. (2018) 143:3143–54. 10.1002/ijc.31657 29978472

[10] Ransohoff JD Wei Y Khavari PA. The functions and unique features of long intergenic non-coding RNA. Nat Rev Mol Cell Biol. (2018) 19:143–57. 10.1038/nrm.2017.104 29138516 PMC5889127

[11] Wang ZK Yang L Wu LL Mao H Zhou YH Zhang PF. Long non-coding RNA LINC00261 sensitizes human colon cancer cells to cisplatin therapy. Braz J Med Biol Res. (2017) 51:e6793. 10.1590/1414-431x20176793 29267503 PMC5731330

[12] Yang L Tang Y He Y Wang Y Lian Y Xiong F. High expression of LINC01420 indicates an unfavorable prognosis and modulates cell migration and invasion in nasopharyngeal carcinoma. J Cancer. (2017) 8:97–103. 10.7150/jca.16819 28123602 PMC5264044

[13] Gao X Wen J Gao P Zhang G Zhang G. Overexpression of the long non-coding RNA, linc-UBC1, is associated with poor prognosis and facilitates cell proliferation, migration, and invasion in colorectal cancer. Onco Targets Ther. (2017) 10:1017–26. 10.2147/OTT.S129343 28260919 PMC5328601

[14] Yu T Shan TD Li JY Huang CZ Wang SY Ouyang H. Knockdown of linc-UFC1 suppresses proliferation and induces apoptosis of colorectal cancer. Cell Death Dis. (2016) 7:e2228. 10.1038/cddis.2016.124 27195675 PMC4917661

[15] Zhou P Sun L Liu D Liu C Sun L. Long non-coding RNA lincRNA-ROR promotes the progression of colon cancer and holds prognostic value by associating with miR-145. Pathol Oncol Res. (2016) 22:733–40. 10.1007/s12253-016-0061-x 27071407

[16] Marín-Béjar O Mas AM González J Martinez D Athie A Morales X. The human lncRNA LINC-PINT inhibits tumor cell invasion through a highly conserved sequence element. Genome Biol. (2017) 18:202. 10.1186/s13059-017-1331-y 29078818 PMC5660458

[17] Virgilio E Giarnieri E Giovagnoli MR Montagnini M Proietti A D'Urso R. Gastric cancer cells in peritoneal lavage fluid: a systematic review comparing cytological with molecular detection for diagnosis of peritoneal metastases and prediction of peritoneal recurrences. Anticancer Res. (2018) 38:1255–62. 10.21873/anticanres.12347 29491048

[18] Nakamura M Suetsugu A Hasegawa K Satake T Kunisada T Shimizu M. Color-coded imaging distinguishes cancer cells, stromal cells, and recombinant cancer-stromal cells in the tumor microenvironment during metastasis. Anticancer Res. (2018) 38:4417–23. 10.21873/anticanres.12743 30061205

[19] Gong J Zhang H He L Wang L Wang J. Increased expression of long non-coding RNA BCAR4 is predictive of poor prognosis in patients with non-small cell lung cancer. Tohoku J Exp Med. (2017) 241:29–34. 10.1620/tjem.241.29 28077810

[20] Quan Y Song K Zhang Y Zhu C Shen Z Wu S. Roseburia intestinalis-derived flagellin is a negative regulator of intestinal inflammation. Biochem Biophys Res Commun. (2018) 501:791–99. 10.1016/j.bbrc.2018.05.075 29772233

[21] Huang Y Hu Y Jin Z Shen Z. LncRNA snaR upregulates GRB2-associated binding protein 2 and promotes proliferation of ovarian carcinoma cells. Biochem Biophys Res Commun. (2018) 503:2028–32. 10.1016/j.bbrc.2018.07.152 30093110

[22] Sun L Xue H Jiang C Zhou H Gu L Liu Y. LncRNA DQ786243 contributes to proliferation and metastasis of colorectal cancer both *in vitro* and *in vivo*. Biosci Rep. (2016) 36:e00328. 10.1042/BSR20160048 26934980 PMC4859087

[23] Zhou J Li X Wu M Lin C Guo Y Tian B Knockdown of long noncoding RNA GHET1 inhibits cell proliferation and invasion of colorectal cancer. Oncol Res. (2016) 23:303–9. 10.3727/096504016X14567549091305 PMC7838607 27131316

[24] Zhai HY Sui MH Yu X Qu Z Hu JC Sun HQ. Overexpression of long non-coding RNA TUG1 promotes colon cancer progression. Med Sci Monit. (2016) 22:3281–7. 10.12659/MSM.897072 27634385 PMC5027858

[25] Yue B Cai D Liu C Fang C Yan D. Linc00152 functions as a competing endogenous RNA to confer oxaliplatin resistance and holds prognostic values in colon cancer. Mol Ther. (2016) 24:2064–77. 10.1038/mt.2016.180 27633443 PMC5167786

[26] Shen P Pichler M Chen M Calin GA Ling H To wnt or lose: the missing non-coding linc in colorectal cancer. Int J Mol Sci. (2017) 18:E2003 10.3390/ijms18092003 28930145 PMC5618652

[27] Anaya J OncoLnc: linking TCGA survival data to mRNAs, miRNAs, and lncRNAs. PeerJ Comp Sci. (2016) 2:e67 10.7717/peerj-cs.67

[28] Chen Y Liu L Li J Du Y Wang J Liu J. Effects of long noncoding RNA (linc-VLDLR) existing in extracellular vesicles on the occurrence and multidrug resistance of esophageal cancer cells. Pathol Res Pract. (2019) 215:470–77. 10.1016/j.prp.2018.12.033 30606658

[29] Li C Zhao Z Zhou Z Liu R. Linc-ROR confers gemcitabine resistance to pancreatic cancer cells via inducing autophagy and modulating the miR-124/PTBP1/PKM2 axis. Cancer Chemother Pharmacol. (2016) 78:1199–207. 10.1007/s00280-016-3178-4 27785603

[30] Sui H Fan ZZ Li Q. Signal transduction pathways and transcriptional mechanisms of ABCB1/Pgp-mediated multiple drug resistance in human cancer cells. J Int Med Res. (2012) 40:426–35. 10.1177/147323001204000204 22613403

[31] Breier A Gibalova L Seres M Barancik M Sulova Z. New insight into p-glycoprotein as a drug target. Anti-Cancer Agents Med Chem. (2013) 13:159–70. 10.2174/187152013804487380 22931413

[32] Wang Z Li Y Ahmad A Azmi AS Banerjee S Kong D. Targeting notch signaling pathway to overcome drug resistance for cancer therapy. Biochim Biophys Acta. (2010) 1806:258–67. 10.1016/j.bbcan.2010.06.001 20600632 PMC2955995

[33] Farah E Li C Cheng L Kong Y Lanman NA Pascuzzi P. NOTCH signaling is activated in and contributes to resistance in enzalutamide-resistant prostate cancer cells. J Biol Chem. (2019) 294:8543–54. 10.1074/jbc.RA118.006983 30940724 PMC6544854

[34] Qiu S Deng L Bao Y Jin K Tu X Li J. Reversal of docetaxel resistance in prostate cancer by Notch signaling inhibition. Anticancer Drugs. (2018) 29:871–79. 10.1097/CAD.0000000000000659 29944470

